# Fungal infection monitoring on corneal epithelium ex vivo model and its collection over polyethersulfone membrane for detecting *Candida albicans* and *Aspergillus fumigatus*

**DOI:** 10.1007/s00430-025-00820-8

**Published:** 2025-02-07

**Authors:** Sarp Orgul, Angela Gómez Bedoya, Víctor Felipe Pérez, Daniella R. Mora, Alfonso L. Sabater, Darlene Miller, Miguel Holgado

**Affiliations:** 1 Department of Ophthalmology, Ocular Surface Center, Bascom Palmer Eye Institute, University of Miami Health System, Miami, FL USA; 2 Multiplexed Molecular Diagnostic S.L.Calle Munner 8, Barcelona, 08022 Spain; 3 Beauty of Sight Eye Bank, University of Miami Health System, Miami, FL USA; 4 Department of Ophthalmology: Ocular Microbiology Research Laboratory, Bascom Palmer Eye Institute, University of Miami Health System, Miami, FL USA; 5 Optics, Photonics and Biophotonics Group, Centre for Biomedical Technology, Campus de Montegancedo Universidad Politécnica de Madrid, Pozuelo de Alarcón, Madrid, 28223 Spain; 6 Group of Organ and Tissue on-a-chip and In-Vitro Detection, Health Research Institute of the Hospital Clínico San Carlos, IdISSC. C/ Profesor Martín Lagos s/n, 4ª Planta Sur, Madrid, 28040 Spain; 7 Applied Physics and Materials Engineering Department, Escuela Técnica Superior de Ingenieros Industriales, Universidad Politécnica de Madrid, C/ José Gutierrez Abascal, 2, Madrid, 28006 Spain

**Keywords:** Aspergillus fumigatus, Candida albicans, Infectious keratitis, In-vitro detection system, Immunofluorescence, Translational research in fungal infectious

## Abstract

In this paper we report a human ex vivo model of *Aspergillus fumigatus* and *Candida albicans* infection of the cornea. We demonstrate the ability to monitor fungal growth on the cornea through optical and immunofluorescence microscopy. Additionally, we establish a method for collection of fungal antigens in polyethersulfone (PES) membrane-based sample collectors from the surface of fungal inoculated corneas. Immunofluorescence microscopy was performed directly on both corneal tissue and PES membrane sample collectors. For the latter case, we devised an original ad-hoc method by attaching the membranes used for the collection on standard glass slides, which we call glass slide KIT. This enabled easy handling and improved efficiency for specifically recognizing the corresponding fungal infections by simple immunoassay protocols. As a result, we firstly observe the ability to monitor fungi in corneal tissue, and secondly demonstrate the adsorption of fungal antigens onto PES membranes. Thus, we report for the first time the specific detection of fungal corneal infections in easy to use PES membrane based glass slide KITs through simple immunofluorescence, as an alternative to microbiological culture.

**Supplementary Information:**

The online version contains supplementary material available at 10.1007/s00430-025-00820-8.

## Introduction

Fungal keratitis, though an uncommon cause of corneal infections in northern regions, remains an important cause of vision loss worldwide [1, 2]. *Candida*, *Aspergillus*, and *Fusarium* make up the most common etiologies depending on region [1, 3, 4]. While *Aspergillus* demonstrates filamentous growth in vitro and in vivo, *Candida* is capable of replicating in its yeast, pseudohyphal, and hyphal forms [5]. Hyphal replication of *Candida* is observed during infection, associated with tissue invasion, and causes increased pathogenicity. Concentrations of proteins, antigens, and markers vary depending on fungal morphology. These differences manifest clinically during corneal infection [6], therefore development of accurate ex vivo models is especially important. Importantly, *Candida* infections often require distinct therapeutic management with *Candida* keratitis being treated with either voriconazole or amphotericin B while other fungal keratitis being managed with natamycin as first line [7]. As a result, early identification of *Candida* keratitis can allow rapid intervention with superior therapies. In this study we present an ex vivo model of fungal keratitis and design a novel system for translation into clinic allowing for rapid diagnosis of fungal corneal infections.

Various ex vivo models of fungal keratitis exist, each with its own associated limitations. One common model involves inoculation of corneal tissue through intrastromal injection of inoculum [8]. Though this technique allows analysis of stromal infiltration and mycelium formation inside corneal tissue, it may not replicate fungal infection of the superficial corneal epithelium. Analysis of the superficial cornea gains special importance in investigations of dimorphic fungi such as *Candida*. Alternatively, others describe ex vivo models using rings which isolate fungal inoculum on the corneal epithelium [9–11]. Due to corneal curvature, inoculation in this manner remains challenging, requiring specially designed devices.

Current detection systems utilized for identification of fungal corneal infections include, microbiological culture, and polymerase chain reaction (PCR) [12]. Other methodologies like potassium hydroxide and calcofluor white staining can identify presence of fungal elements, however, these techniques are highly non-specific unlike PCR or immunofluorescence based alternatives [13]. Nevertheless, differentiation of fungal infections from non-fungal infections can be especially useful for clinical decision making. In-vivo confocal microscopy has also gained recent traction for diagnosis of fungal keratitis though these techniques require skilled microscopy interpretation, expensive equipment, and still remain non-specific [13].

While corneal scrape with culture remains the gold standard in diagnosis of corneal infection, lengthy culture time remains the main limitation. PCR on the other hand, comes with high costs and the potential for false positives [12]. Fast, accurate diagnosis allows rapid detection and rapid treatment of fungal keratitis, which is associated with improved visual outcomes [6]. As such, other fluorescence based [14] and nucleic acid based [15, 16] diagnostics are being investigated.

More specifically, immunoassay technologies have gained recent relevance in clinical diagnosis both in the field of ophthalmology and more broadly in medicine [17–21]. Enzyme-linked immunosorbent assay (ELISA) can be considered a gold standard laboratory technique providing reliable, accurate and specific detection of target biomarkers present in biological samples. ELISA based techniques have been applied for detection of non-fungal pathogens on the ocular surface [18–21] and for detection of fungal pathogens in serum and sputum [17]. Immunofluorescence microscopy is another relevant technique reported for fungal detection [22, 23] and for localization of a wide range of antigens in different types of samples including ear swabs collected for otomycosis [23]. Fungal identification on the corneal surface, however, is a challenge due to the difficulty of biological sample collection, especially because of the deep, infiltrative nature of fungal growth. Other studies have demonstrated detection of non-fungal infectious antigens in ocular tears with success, though these systems are limited by non-standardized tear collection techniques [20, 24]. While these technologies have been applied broadly in ophthalmology, to the best of our knowledge, there has been limited clinical application of immunofluorescence or ELISA based diagnostic technologies for detection of fungal keratitis. Additionally, some detection techniques described in literature are limited by complex protocols and cost effectiveness. Fluorescence based detection systems have been successfully employed in a number of studies involving corneal scrape samples in both animal models and in human patients allowing the identification of both bacterial and fungal keratitis. These studies underline the immense potential of fluorescence based diagnostics in the setting of corneal infections [25–27].

PES membranes have previously been used for collection of ocular surface samples in isolation of tear microRNA [28], tear protein [29], and conjunctival cells [30]. These membranes have become standard use in sample collection methodologies. PES membrane based ocular sampling devices such as the EYEPRIM (OPIA Technologies, Paris, FR) are commercially available and approved for clinical use. To our knowledge PES membranes have also not yet been used for ocular infection detection. Due to their strong ability to absorb protein we predict that PES membrane will also recover biomarkers necessary for fungal detection.

**Fig. 1:**
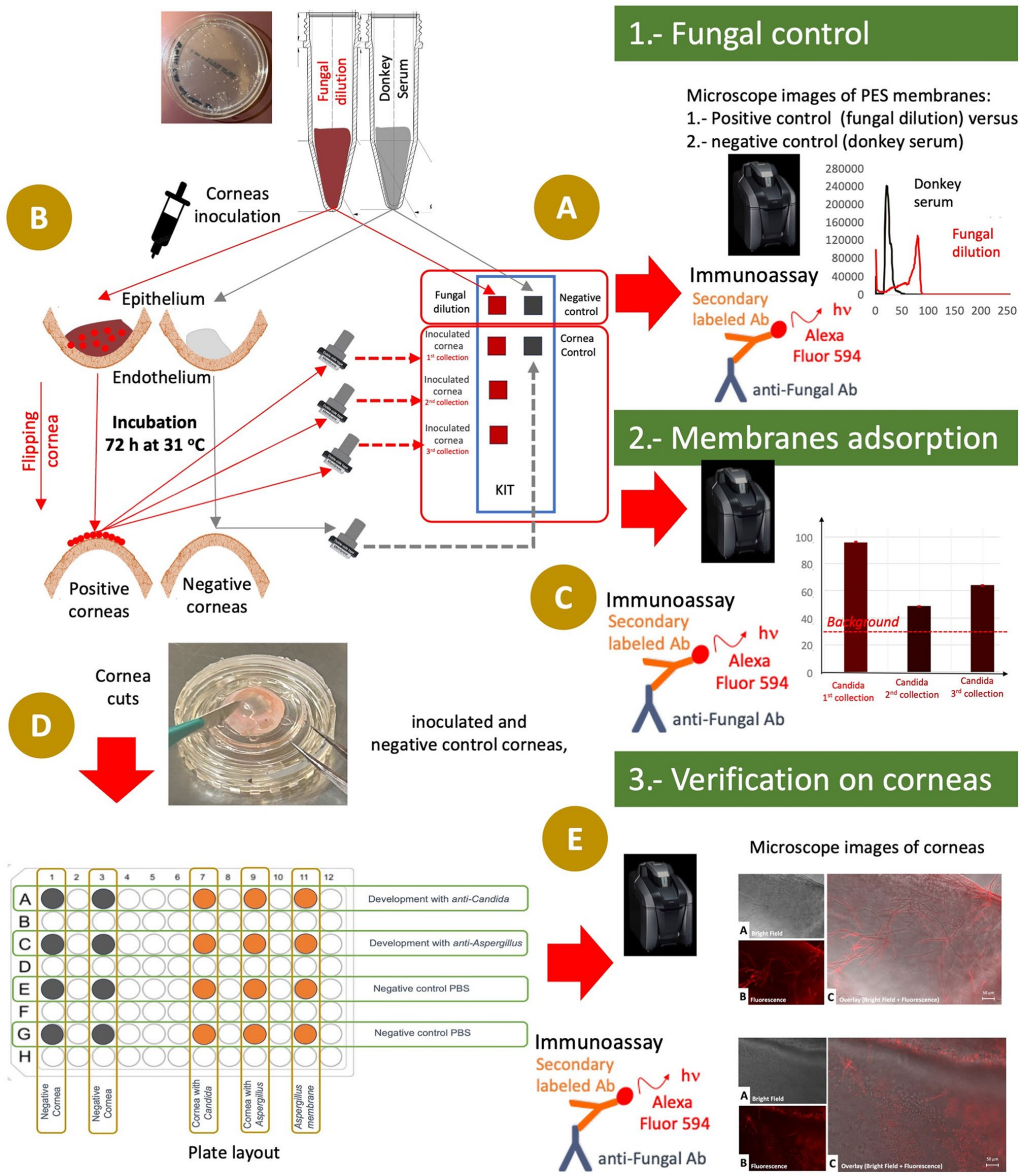
Experimental flow chart. **A)** First step to control that the fungal dilution, we call this positive and negative control because this dilutions are directed placed on the PES membranes. The results are obtained by fluorescence immunoassay, and this is a step to control the fungal dilution. **B)** Second step to flip the corneas and inoculate with the fungal dilutions carrying out in parallel a negative cornea and incubate 72 h at 31 °C. **C)** Third step to proceed to collect with the PES membranes from the epithelium corneas surface. These membranes are glass specimen (the KIT) and evaluate the membranes adsorption by immunofluorescence. **D)** Fourth step to cut the corneas in pieces and put these in a plate to check and compare the results obtained with the PES membranes. **E)** Final step to verify the results by immunofluorescence directly in the corneas we used for the collection in order to observe and compare the results

In this work, we develop and report a platform for investigating fungal keratitis through an ex vivo model. We present immunofluorescent staining based microscopy procedures for monitoring *Candida albicans* and *Aspergillus fumigatus* infection on corneal epithelium ex vivo (Fig. 1). We also demonstrate collection of fungal antigens by PES membranes, which can be attached on glass slides for viewing. For this technique, we establish original glass slide KITs to permit specific recognition of *Candida albicans* and *Aspergillus fumigatus* from the infected corneas by simple immunofluorescence protocols. We propose that these immunofluorescence based glass slide KITs as an easy to use and quicker alternative to microbiological culture based fungal identification and have potential translation to clinical use. The application of this detection methodology to ophthalmic care can have a positive impact on clinical practice and allow early detection of corneal fungal infections resulting in improved visual outcomes for patients.

## Materials and methods

In Fig. 1 can be observed the flow chart showing the whole experimental process from (1) the fungal control, (2) the adsorption of the PES membranes and (3) Verification on the results in the corneas where we proceeded to collect the samples by the PES membranes.

### Corneal acquisition and preparation

Experiments were conducted in accordance with the tenets of the Declaration of Helsinki for biomedical research involving human tissue. A total of six human donor research quality corneas were acquired from the Beauty of Sight Eye Bank (Miami, FL) with each cornea assigned to a different condition. Each cornea was inverted so that the epithelial face had concave up orientation, creating a corneal epithelial pit (Fig. 1A). Prior to acquisition, all corneas were stored in standard corneal storage vials with Optisol-G at 4 °C per eye banking protocol. The vials were filled with culture media composed of CTS OptiMem I (Gibco, Carlsbad, CA), 2% fetal bovine serum, 10,000 units/mL penicillin, and 10,000 µg/mL streptomycin. Culture media was filled up to the point of contact with the inferior side of the cornea to ensure isolation of fungal inoculate. The corneal epithelial surface was not in contact with the corneal culture media.

### Corneal inoculation

*Candida albicans* and *Aspergillus fumigatus* isolates were cultured on Sabouraud dextrose agar containing 50.0 mg of chloramphenicol and 5.0 mg gentamicin for 72 h. A 0.5 McFarland solution of *C. albicans* and *A. fumigatus* equivalent to ~ 10^6^ CFU/mL was prepared by dilution in Dulbecco’s Phosphate Buffered Saline (DPBS). Fungal concentration of the stock solution was confirmed through culture and colony counting using a hemocytometer.

**Fig. 2:**
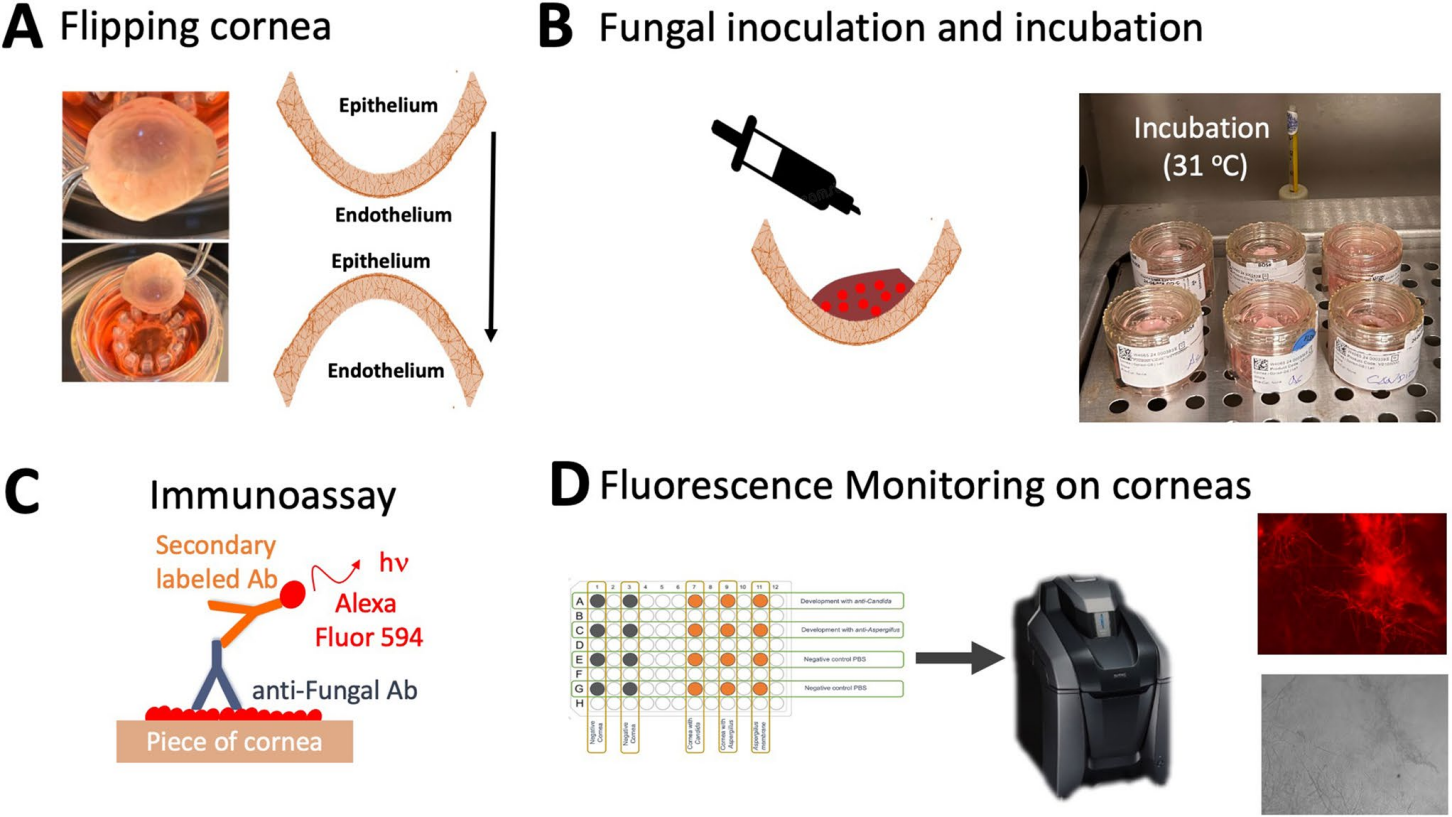
Scheme of corneal preparation (picture), inoculation, immunoassay and observation

Corneas were acquired in donor pairs. Corneas originating from the left eye were inoculated with a fungal suspension while corneas originating the right eye were used as negative controls and inoculated with Phosphate-Buffered Saline (PBS). For the treatment corneas, at the center of the corneal pit and on the epithelial aspect of the cornea, 10 µL of the stock fungal solution was pipetted. For control corneas 10 µL of PBS was pipetted. The resulting preparation was cultured at 31 °C with 5% CO_2_ for a period of 48 h (Fig. 2A and B).

### PES membrane and collection of the fungal samples

**Fig. 3:**
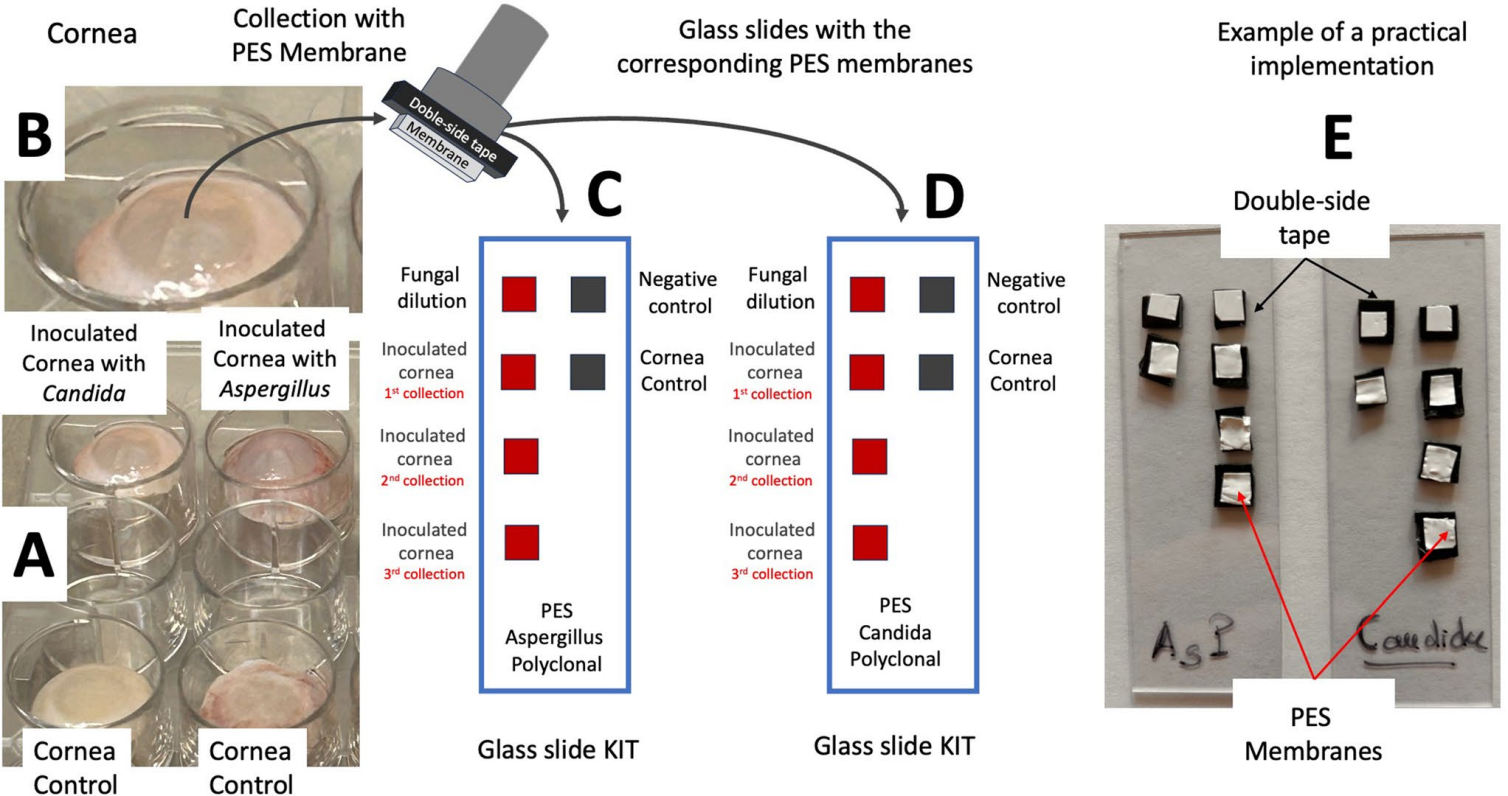
**A**.- Plate with the ex-vivo corneas. **B**.- Detail of a cornea where the collection with the PES membrane takes place. **C**.- Layout of the glass slides KITs containing membranes adhered with double-sided tape for Aspergillus evaluation. **D**.- Layout of the glass slides KITs containing membranes adhered with double-sided tape for Candida evaluation. **E**.- Practical implementation of the glass slide KITs. It is in these KITs where the immunoassay is carried out

At 48 h of culture, corneas were removed from their chamber, and inverted resulting in the original corneal orientation (Fig. 1B) with the epithelium facing up. The 48 h time point was chosen in accordance with prior literature [8]. The corneas were placed in a cell culture plate and on top of a Millicell cell culture insert (MilliporeSigma, Burlington, MA) as scaffolding. The corneas were kept in this orientation for two minutes to dry the epithelial surface for sample collection (Fig. 3A). PES membranes (Ref. SF15161 Tisch scientific, Cleves, OH) were used for sampling of the corneal surface. An immunoassay was applied for specific detection of fungal presence. The PES membranes were cut to obtain a dimension of approximately 4 × 4 mm^2^ and adhered to a sample collection stick using double sided tape. This size permits easy collection of the sample from the cornea. After sample collection, the PES membranes together with double sided tape were removed from the sample collection stick and adhered to a standard glass slide KIT. The resulting KIT allowed for monitoring the fluorescence on each membrane individually (Fig. 3C-E).

For all cornea, the samples were collected through placement of the PES membrane on the corneal epithelial surface with gentle pressure for three seconds (Fig. 3B). For negative control corneas, only one sample was collected. In corneas with fungal inoculation, three consecutive samples were collected from the same location on each cornea. This process was done for the *Candida albicans* inoculated cornea and its negative control cornea. Then also conducted for the *Aspergillus fumigatus* inoculated cornea and its negative control cornea. The PES membranes were removed from the sample collectors and secured to a glass slide with the orientation depicted (Fig. 3C-E). We call this glass slide KIT.

Two additional membranes were placed onto the glass slide KIT for each fungus. The first additional membrane was used as a positive control and 10 µL of *Candida albicans* or *Aspergillus fumigatus* stock suspension were pipetted onto the PES membrane. The second membrane acted as a donkey serum based negative control. All samples were kept at 4 °C for same day processing.

### *In situ* fluorescence monitoring on the membranes through immunoassay

An immunoassay methodology was applied to stain the collected samples. The same bioreagents used for the monitoring in the corneas were also used for monitoring the membranes. However, the immunoassay steps were adapted into the following steps: immobilization or sample collection, blocking to avoid the unspecific adsorption, specific recognition with primary antibody, and development with a labeled secondary antibody. This process was previously optimized to ensure a correct specific detection of the collected samples coming from the inoculated ex vivo corneas with *Aspergillus fumigatus*, *Candida albicans*, and non inoculated corneas as negative control on the PES membranes. Also a positive and negative control was considered to ensure that the results of the specific detection of the fungal infections considered were significant.

Figure 3C and D show the different membranes employed for controls and samples collected, positive control (fungal suspension), negative control (donkey serum), cornea control (non-inoculated cornea) and the corresponding collection of the inoculated corneas. For the donkey serum negative controls and for the fungal suspension positive controls the first step was performed by immobilization of the respective solutions onto their PES membranes. In the case of the negative control 5 µL of 1:2 diluted donkey serum was pipetted onto the PES membrane. For the fungal suspension positive control, the first step was conducted by pipetting 5 µL of fungal dilution (10^6^ CFU/mL) onto its respective membranes (See Fig. 3C).

For all other samples, the first step was conducted through PES sample collection from their respective corneas and by transfer of the PES membrane onto the glass slide KITs in their respective positions (Fig. 3B-C).

For this immobilization collection step an incubation time of 1 h at 37 ºC was used followed by a washing step involving submerging of the KITs into a 50 mL Falcon tube containing ultrapure water and gentle shaking for 10 min.

After immobilization, a blocking step was performed to avoid the nonspecific adsorption. This was carried out by pipetting 5 µL of donkey serum on all membranes of the KIT. The KITs were then incubated for 30 min at 37 ºC in a humid chamber and a washing step of 10 min was conducted as described before.

For fungal recognition, the same specific polyclonal antibodies against *Aspergillus fumigatus* and *Candida albicans* mentioned prior were applied by pipetting 5 µL of the corresponding antibodies in all membranes of the KITs. An incubation of 3 h at 37 ºC in a humid chamber was performed followed by 10 min of washing with shaking.

Finally, the same secondary antibody mentioned prior (goat anti-Rabbit antibody conjugated with Alexa Fluor 594) was used for developing the cornea and 5 µL of the secondary was applied to each membrane of the KIT. The KITs were incubated for 1.5 h at 37 ºC in humid conditions. The assay was concluded with a final 10 min washing step under shaking conditions. The KITs were dried with air prior to imaging.

The resulting KITs slides were imaged using a Keyence BZ-X810 (Keyence, Osaka, JP) fluorescent microscope which conditions were optimized for Candia and Aspergillus resulting in standard resolution at 0.5 s acquisition time for Candida and high resolution with an acquisition time of 1 s for Aspergillus. It is very important to remark that these PES membranes were of a size about 4 × 4 mm^2^ and the microscope objective employed for the evaluation of each membrane was 4x, with a numerical aperture of 0.2, a working distance of 20 mm that permit a field of view of 3.623 × 2.728 mm2, a size similar to the whole size of the corresponding membranes.

The images were centered around the PES membranes and were acquired using the same exposure time. For analysis, every red pixel in the image was counted and grouped by red tone value. Mean red tone for each image was calculated and the standard error of this mean was computed.

### *In situ* fluorescence monitoring on the corneas through immunoassay

The resulting corneas were preserved using 4% paraformaldehyde diluted in PBS for six hours. They were then transferred into a solution of 30% sucrose for deswelling until corneas were observed to have dropped to the bottom of their respective solutions. Each cornea was then divided into four quadrants (Fig. 1D). For inoculations of *Aspergillus fumigatus*, the superficial mycelium overlying the cornea was also separately removed, divided into quadrants, and imaged.

Each tissue was specifically recognized with primary antibodies for *Candida albicans* and *Aspergillus fumigatus* and incubated. Immunoassays were carried out to monitor the corresponding fungal infections on the corneas. Specific rabbit IgG polyclonal antibodies against *Aspergillus fumigatus* (ThermoFisher Scientific Ref. PA1-7202, Waltham, MA) and against *Candida albicans* (ThermoFisher Scientific Ref. PA1-7206, Waltham, MA) were prepared at a concentration of 40 µg/mL, pipetted directly into the plates completely covering the corneal cuts, and incubated for 3 h with humidity at 37 °C. After incubation, the corneas were washed with DPBS. Next, the corneas were developed with the secondary goat anti-rabbit antibody conjugated with Alexa Fluor 594 (ThemoFisher Scientific, Waltham, MA) with excitation from 561 nm to 594 nm and emission in the red spectral range. A concentration of 4 µg/mL was pipetted completely covering the cornea and incubated for 1.5 h at 37 ºC. The cornea was washed with DPBS. For all corneas, one set of corneal cuts was kept in DPBS to act as a baseline negative control. This process was implemented under the optimization of previous experiments to ensure a correct monitoring of the infection on ex vivo corneas and to be sure of the correct infection of the corneas with *Aspergillus fumigatus* and *Candida albicans* in comparison with the uninfected corneas used as negative control. We monitored the whole pieces of cornea with the different microscope objectives observing homogeneity and we concluded that there were not significant differences of the inoculation.

After this, the corneas were ready for fluorescent monitoring. The resulting tissues were imaged using a Keyence BZ-X810 (Keyence, Osaka, JP) fluorescence microscope (Fig. 2C-D) with microscope objectives 10x (field of view 1.449 × 1.091 mm^2^) and 20x (field of view 0.725 × 0.546 mm^2^). Images for 4x (field of view 3.623 × 2,728 mm^2^) can be observed in supplementary information.

### Statistical analysis

Fluorescent images were acquired and centered around the membranes placed on the KITs. To compare the different experimental outcomes, Fig. 8 (for *Candida albicans*) and Fig. 9 (for *Aspergillus fumigatus*) shows the results obtained in the corresponding glass slide KITs, where the different PES membranes were placed: A.-negative versus positive controls (dilution of donkey serum versus fungal dilution), B.- Cornea negative control (cornea without fungal inoculation) versus cornea positive control (cornea with fungal inoculation) and C.- different fungal collections in infected corneas. For each fluorescent membrane image, a histogram was produced. In other words, the number of pixels with each possible red tone (from 0 to 255) was counted and graphed. Next, the statistical expected value of the red tone or average signal was calculated for each image as follows: We firstly obtained the corresponding histograms of the immunofluorescence experimental results observed in the different PES membranes or experiments. The total number of points of the image analyzed for each membrane were 2,764,800 for obtaining these corresponding histograms in each PES membrane. The different levels of red intensity, or red tone, obtained in the image of each PES membrane is developed by using a secondary goat anti-rabbit antibody conjugated with Alexa Fluor 594, whose fluorescence emission is in red tone. Thus, the red tone value obtained was divided in 255 levels to construct the histograms for each experiment. This can be observed in the corresponding Fig. 8 (for *Candida albicans*) and 9 (for *Aspergillus fumigatus*) of the manuscript. Thus, for each histogram we obtained the statistical expected value (weighted red tone average, $\:E\left(x\right)=\stackrel{-}{x}$) of the red tone, or in other words, the average red value of each PES membrane or experiment carried out. This expected value mathematically defined as the red tone average of each experiment, is calculated by the equation:

$$\:E\left(x\right)=\sum\:{x}_{i}{\:p}_{i}$$ Eq. 1

Where $\:{x}_{i}$ is the red tone from i = 0 to i = 255 levels represented in the horizontal axis (x-axis, or abscissa) in each of the histograms of Figs. 8 and 9, which in turn represent the results of each of the experiments performed. It should be noted that each membrane corresponds to its own experiment. Thus, $\:{\:p}_{i}$ is the weighted red intensity (y-axis or ordinate axis of the histogram) calculated as the intensity at each red tone, where $\:{y}_{i}$ is the number of pixels for each red tone, divided by the total intensity. This intensity is obtained in the histogram as the number of pixels observed in each corresponding red tone. Therefore, we obtained $\:{\:p}_{i}$ as follows:

$$\:{p}_{i}=\frac{{y}_{i}}{\varSigma\:{y}_{i}}$$ Eq. 2

Finally the standard error of the average or the statistical expected value (weighted red tone average) was calculated to obtain the uncertainty of the measurement for each signal of the image. After the calculation of the expected value the variance of the red tone is calculated by the equation:

$$\:Var\:\left(x\right)=\varSigma\:{\:\left({x}_{i}-\stackrel{-}{x}\right)}^{2}\:{p}_{i}\:$$ Eq. 3

and thus, it can be easily calculate the experimental standard deviation (s) as the square root of the variance ($\:Var\:\left(x\right)$. The standard uncertainty *u(x)* of the average red tone *(*$\:\underset{\_}{x}$*)* or the statistical standard error can be calculated following the recommendation of the Guide to the expression of Uncertainty in Measurement (GUM) as:

$$\:u\left(\stackrel{-}{x}\right)=\frac{s\:\left(x\right)}{\sqrt{i}}$$ Eq. 4

where the estimated $\:\mu\:$ is $\:\underset{\_}{x}$ corresponding to the average red tone $\:E\left(x\right)$ and “*i”* is the number of statistical samples, for this case the 255 red tones considered for the constructed histogram. we can consider the expanded uncertainty as:

$$\:\left(\stackrel{-}{x}\right)=\:k\cdot\:\:u\:\left(\stackrel{-}{x}\right)$$ Eq. 5

where k is the coverage factor. The value of the coverage factor k is chosen on the basis of the level of confidence required and generally, k will be in the range 2 (for an estimated significance level of 95%) to 3 (for a significance level of 99%). We have calculated the error bars for a coverage factor of k = 3, and therefore the estimated significance level is 99% [31]. The resulting values were depicted as bar graphs (Figs. 8 and 9) which values were very small. Differences between average red signal values were considered significant if they had non-overlapping the expanded statistical uncertainty. Moreover, a difference of means test was carried out to ensure the evidence of the reported results.

## Results

### *In situ* fluorescence Monitoring in Corneal epithelium

#### Results in Candidal inoculation

**Fig. 4:**
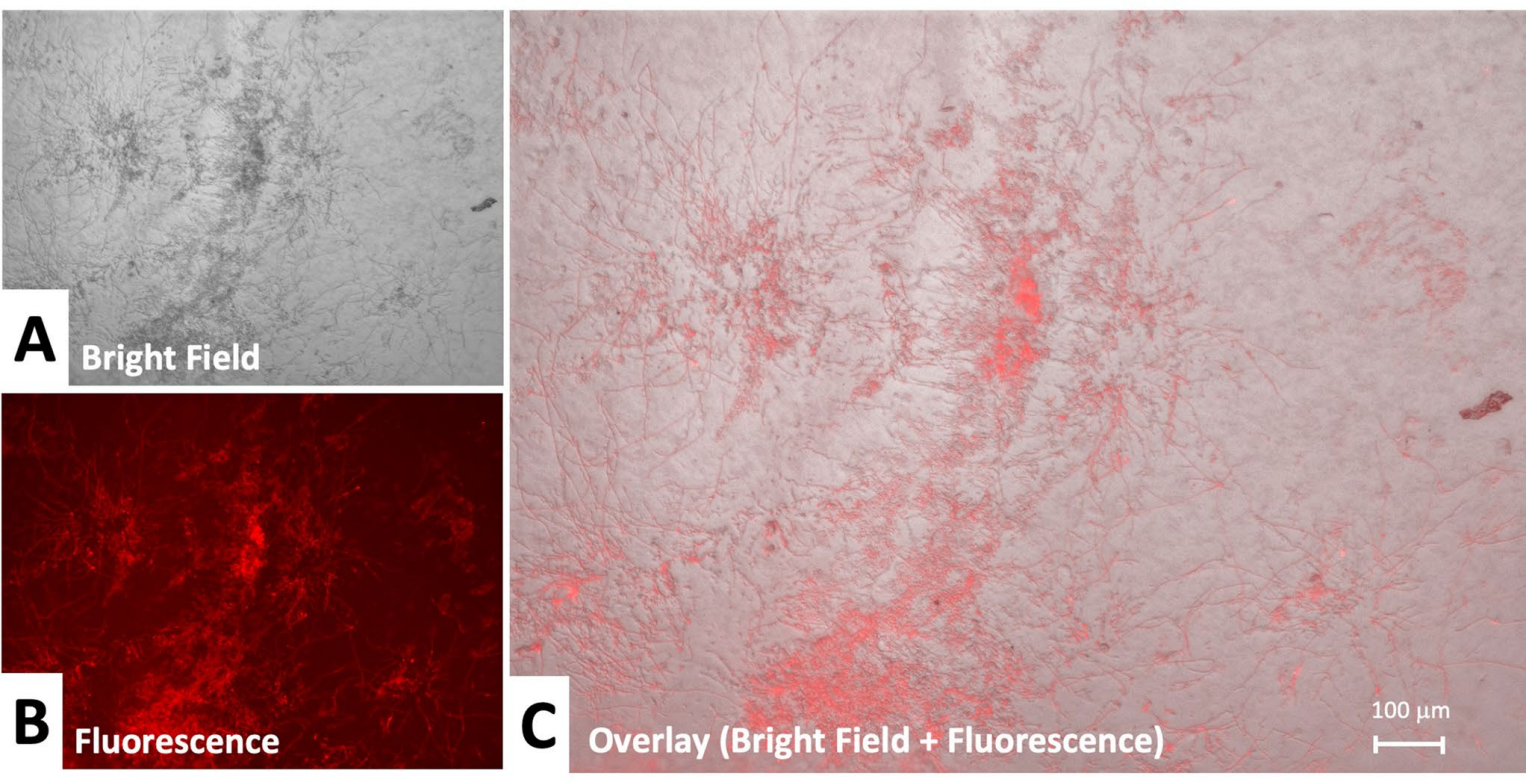
Candida infection in cornea with full focus: (**A**) Bright field 10x, (**B**) Fluorescence under specific anti-candida recognition plus secondary labeled antibody (**C**) Overlay of fluorescence plus bright field. Yeast-like forms and pseudohyphal extension of Candida is evident. Strong fluorescent labeling of yeast and pseudohyphal elements is observed with specific candida recognition. Candida growth is observed largely superficially on the corneal tissue

We first assessed fungal inoculation of the corneas through microscopy. In tissue inoculated with *Candida albicans*, growth of yeasts was observed on the central aspects of the cornea in all corneal quadrants. High density of yeast appeared centrally and density of clusters diminished in the periphery of the cornea. *Candida albicans* growth was mostly observed superficially on the corneal epithelium. In addition, we clearly observed hyphal or pseudohyphal growth of candidal fungus. On labeling with anti-candida antibodies, candidal spores and hyphae were positive for fluorescence (Fig. 4B). Negative control corneal tissue demonstrated no fungal growth on brightfield microscopy and remained without red fluorescence signal.

#### Results in aspergillus inoculation

**Fig. 5:**
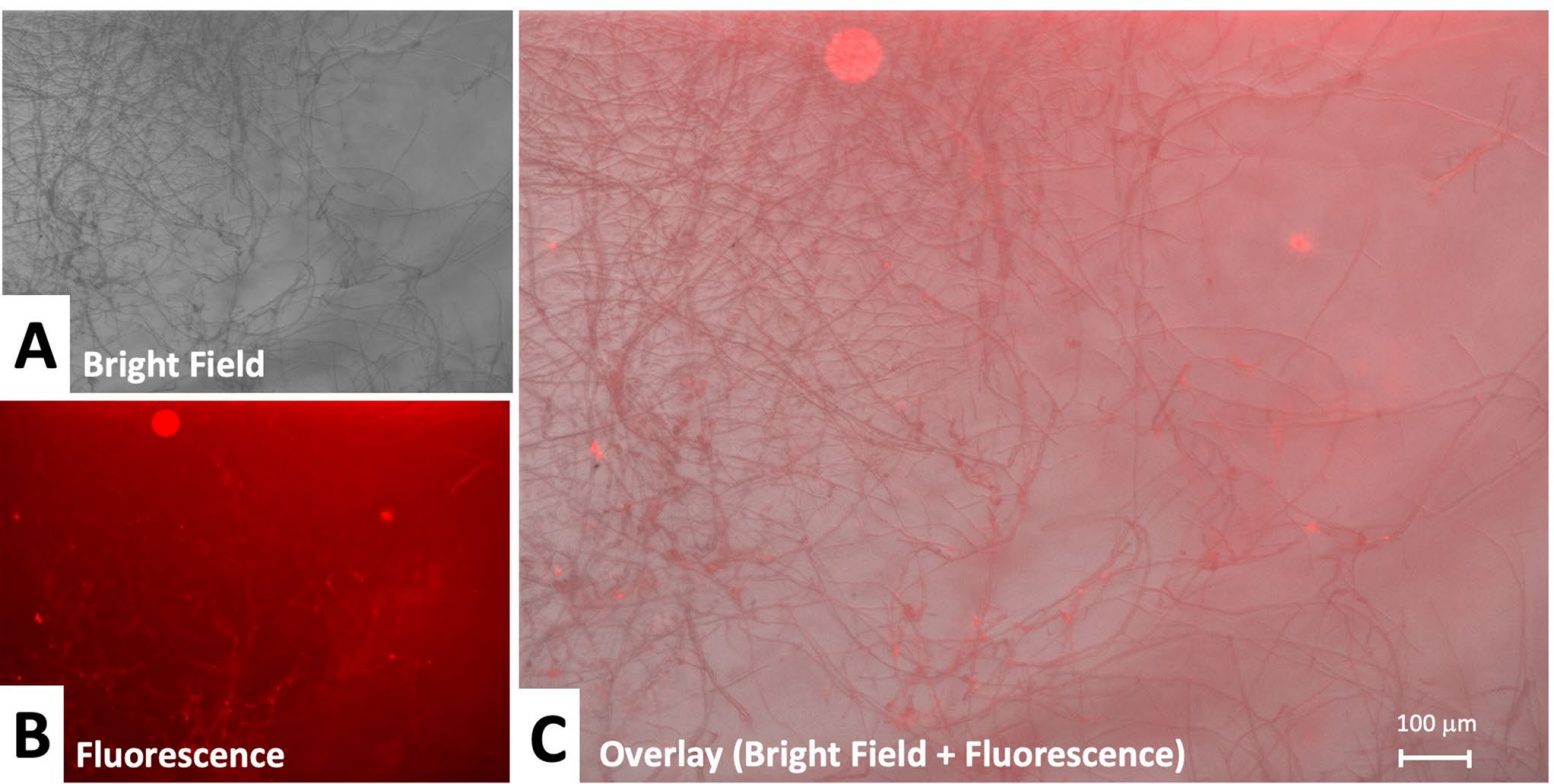
Aspergillus infection in cornea with full focus: (**A**) Bright field 10x, (**B**) Fluorescence under specific anti-candida recognition plus secondary labeled antibody (**C**) Overlay of fluorescence plus bright field. Hyphal extension of Aspergillus is observed. Strong fluorescent labeling of hyphal elements is observed with specific aspergillus recognition. Growth is mainly hyphal in nature

Corneal tissue from corneas inoculated with *Aspergillus fumigatus* contained hypaeated fungi and tissues were covered with a filamentous fungal biofilm, which infiltrated corneal stroma and exhibited a three dimensional pattern of growth (Fig. 5). Hyphal tips were observed superficially on the epithelial aspect of the cornea (Fig. 7). Additionally, superficially exposed hyphal tips of *aspergillus* presented with the strongest labeling. Anti-aspergillus antibodies also exhibited a high degree of reactivity for the *Aspergillus fumigatus* fungal biofilm. Negative control corneal tissue demonstrated no fungal growth on brightfield microscopy and remained without fluorescence.

#### Infection propagation

**Fig. 6:**
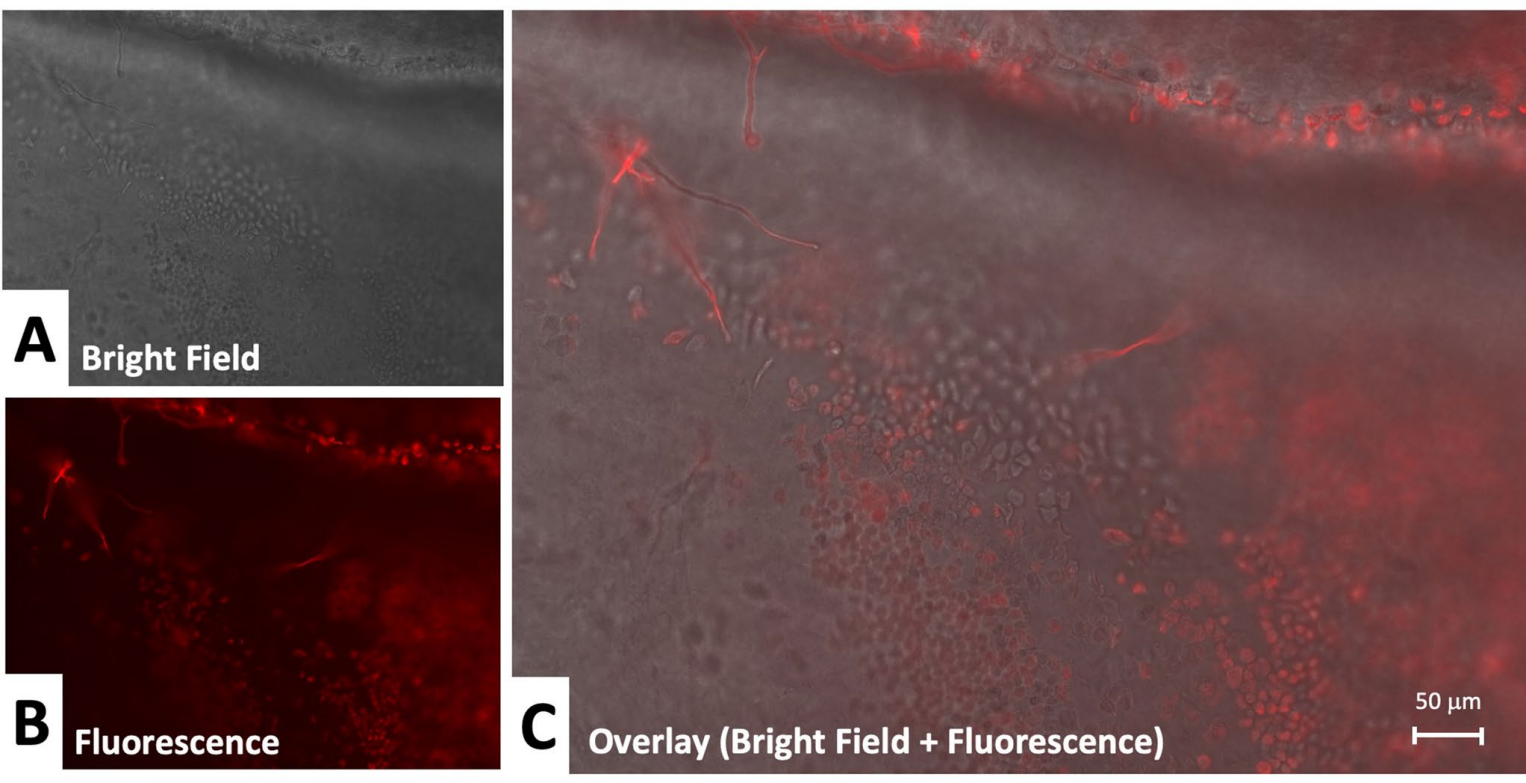
Candida infection propagation in cornea: **A**. Bright field 20x, **B**.- Fluorescence under specific anti-candida recognition plus secondary labeled antibody **C**. Overlay of fluorescence plus bright field. Yeast-like forms and pseudohyphal extension of candida is visible at this higher magnification. Strong fluorescent antibody labeling of both yeast and pseudohyphae elements is observed

**Fig. 7:**
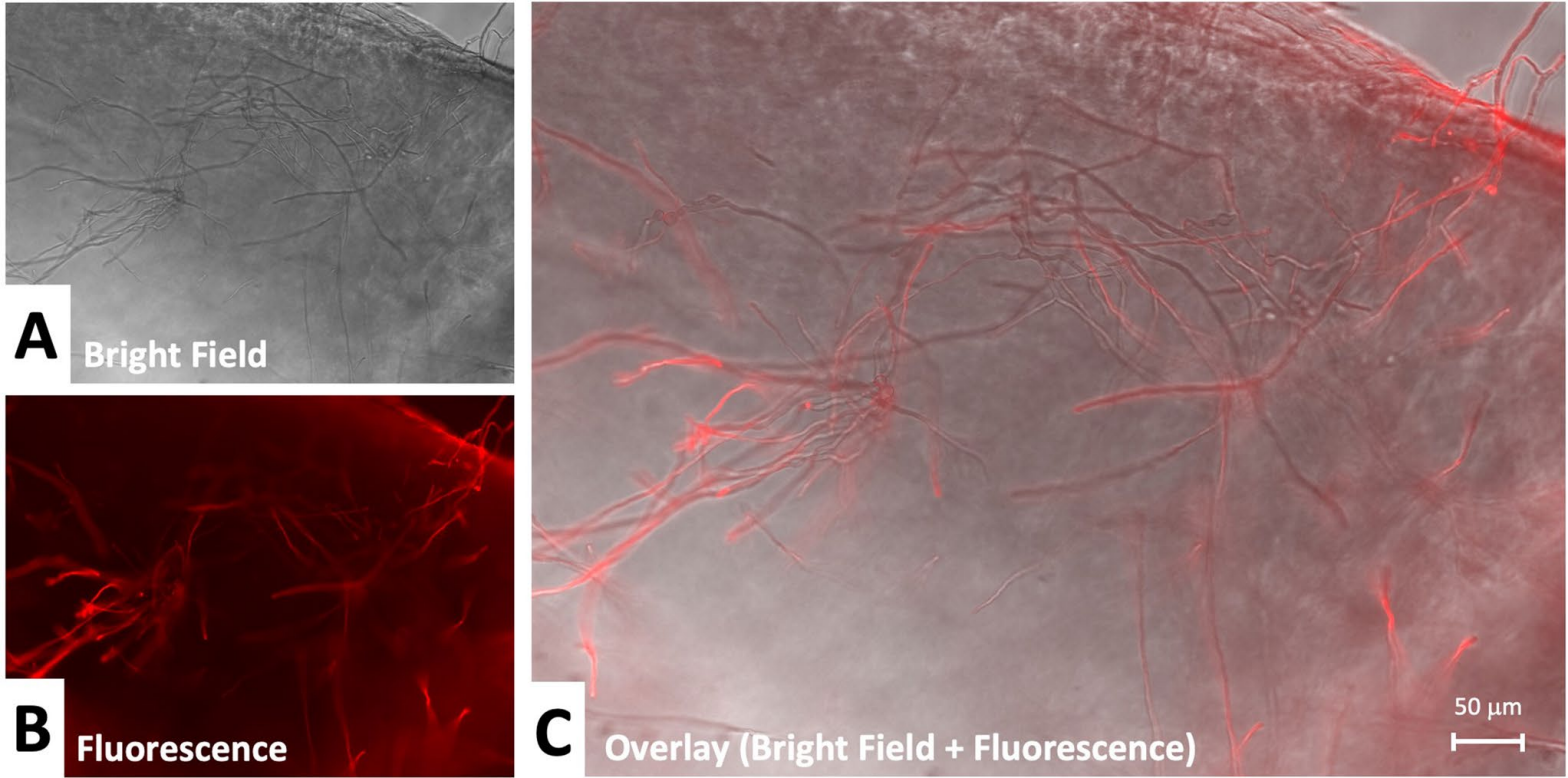
Aspergillus infection propagation in cornea: (**A**) Bright field 20x, (**B**) Fluorescence under specific anti-candida recognition plus secondary labeled antibody (**C**) Overlay of fluorescence plus bright field. Hyphal aspergillus propagation is observed. Strong labeling of superficial hyphal elements is observed with less prominent labeling of aspergillus extending into stroma. The edge of the corneal tissue cut is apparent at the corner of the image

To examine infection propagation in more detail, we used higher magnification. This revealed a clearer candidal hyphal or pseudohyphal pattern of growth (Fig. 6). In comparison to the growth of *aspergillus*, *candida* demonstrated increased spore formation and more superficial distribution. Growth of *aspergillus* remained mainly hyphal and extended into the corneal stroma (Fig. 7). The depth of growth appears to be demonstrated by differences in focal point on microscopy and lack of labeling of stromal components.

### Monitoring of corneal epithelium through PES membranes by immunoassay

#### Results for Candida

**Fig. 8:**
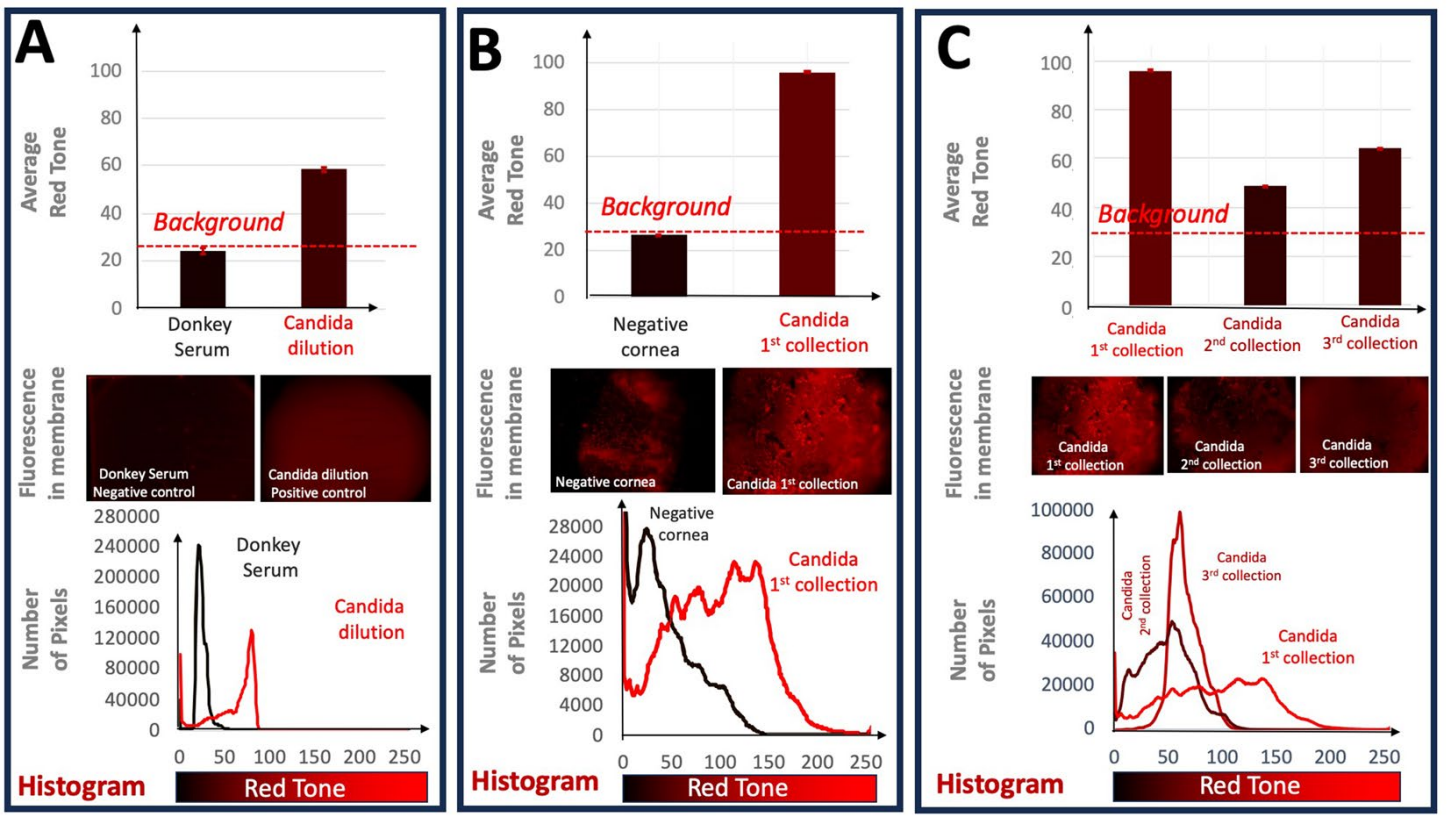
Fluorescence evaluation in the corresponding glass slide KITs for Candida albicans. Red tone in bar graphs corresponds to average red tone observed in each group. **A**.- Comparison between negative control (donkey serum) and Fungal dilution. **B**.- Comparison of specific detection of collected Candida albicans inoculated versus the negative control cornea (non-inoculated). **C**.- Comparison of subsequent collections in the same cornea inoculated with Candida albicans. Error bars are corresponding to the expanded uncertainty for a coverage factor of k = 3

To demonstrate the PES membrane’s ability to collect fungal antigens we first demonstrated the capability of our glass slide KIT to differentiate between Candida albicans fungal suspension and a donkey serum control. We observe a right shifted histogram and higher average red tone (redder) in the candidal sample when compared to the control sample (Donkey serum: 24.233 ± 0.0145; Candida albicans suspension: 58.515 ± 0.046) (Fig. 8A). We next evaluated the ability of the glass slide KIT to properly identify antigens from the Candida albicans inoculated cornea. The collection from the Candida albicans cornea also showed a right shifted histogram and redder average tone when compared to sample collection from the negative control cornea (Negative cornea: 26.793 ± 0.059; Candida albicans cornea: 95.964 ± 0.084) (Fig. 8B). The donkey serum control and negative control cornea demonstrated similar background fluorescence (Donkey serum: 24.233 ± 0.014; Negative cornea: 26.793 ± 0.059). Finally, we compared multiple subsequent sample collections from the Candida albicans cornea. Second and third sample collections resulted in lower average red tone when compared to the first collection (First: 95.964 ± 0.084; Second: 48.489 ± 0.043; Third: 64.153 ± 0.026) (Fig. 8C).

#### Results for aspergillus

**Fig. 9:**
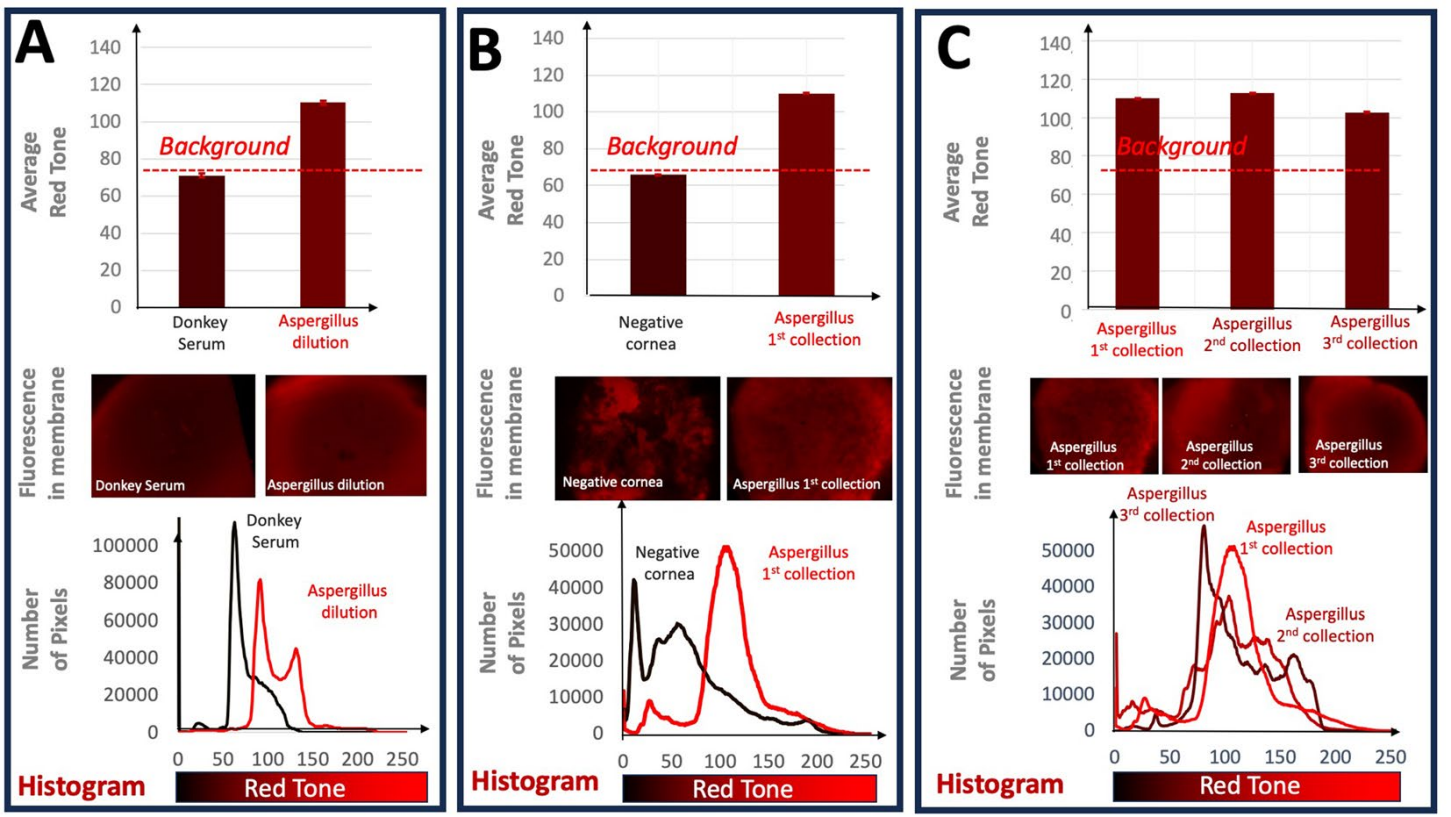
Fluorescence evaluation in the corresponding glass slide KITs for Aspergillus fumigatus. Red tone in bar graphs corresponds to average red tone observed in each group. **A**.- Comparison between negative control (donkey serum) and fungal dilution. **B**.- Comparison of specific detection of collected Aspergillus fumigatus inoculated versus the negative cornea (non-inoculated). **C**.- Comparison of subsequent collections in the same cornea inoculated with Aspergillus fumigatus. Error bars are corresponding to the expanded uncertainty for a coverage factor of k = 3

Evaluation of PES membrane efficacy with collection from *Aspergillus fumigatus* corneas revealed similar results. Again, we demonstrated the PES membrane’s ability to collect *Aspergillus fumigatu*s antigens with *Aspergillus* suspension having redder average tone when compared to the donkey serum control (Donkey serum: 70.971 ± 0.048; *A. fumigatus* suspension: 110.304 ± 0,051) (Fig. 9A). We also observed higher average red tone in the *Aspergillus fumigatus* inoculated cornea when compared to the negative control cornea (Negative cornea: 65.909 ± 0.081; *Aspergillus fumigatus* cornea: 110.129 ± 0.065) (Fig. 9B). Unlike with *Candida albicans*, second and third sample collections revealed similar levels of signal when compared to the first sample collection (First: 110.129 ± 0.065; Second: 112.907 ± 0.063; Third: 102.750 ± 0.072) (Fig. 9C).

## Discussion

In this paper we establish a new technique based on PES membrane sample collectors and immunofluorescence for detection of *Candida albicans* and *Aspergillus fumigatus* in an ex vivo model of corneal infection. We report an immunoassay-based methodology for identification of fungal infection directly on corneal tissue. We implement a new sample collection technique for fungal antigen adsorption onto PES membranes. Finally, we demonstrate the ability to specifically recognize these antigens through the use of our PES membranes based KITs as an alternative to microbiological culture.

Our technique may be most useful with clinical translation allowing rapid in clinic diagnostics allowing for better treatment decisions in the setting of suspected fungal keratitis. Given key differences in management of *Candida* keratitis when compared to other fungal keratitis the distinction of *Candida* infection from non-*Candida* keratitis remains of utmost importance. While natamycin is commonly used as a first line therapy in general for fungal keratitis *Candida* is often managed first line with topical amphotericin-B or voriconazole therapy [7]. In the next stages of this work, this technique could be tested in clinic with patients presenting with fungal infections. Ability to correctly identify fungal corneal infections may allow for earlier selection of optimal therapies.

To our knowledge, this is the first demonstration of the PES membrane’s ability to collect fungal antigens in corneal infection models. We observe that for both *Candida albicans* and *Aspergillus fumigatus*, our methodology consistently detects fungal presence (Figs. 8B and 9B). In addition, successive samplings from the same cornea also remain positive indicating repeatability of our process (Figs. 8C and 9C). Curiously, subsequent samplings of *Candida* resulted in reduced fluorescent signal (Fig. 8C). Conversely, we note that *Aspergillus*, with filamentary growth, can be sampled many times with positive results (Fig. 9C).

We estimate the differences in behavior observed between *Candida* and *Aspergillus* sampling is connected to the differences between filamentary growth and yeast-like replication. *Aspergillus’s* propensity to burrow into corneal stroma may have also resulted in highly fluorescent second and third samplings. *Candida* demonstrated some filamentary and hyphal structures, however, overall *Candida* exhibited more yeast-like growth relative to *Aspergillus*. We predict that on first sampling *Candidal* antigens are removed from the corneal surface. The persistent presence of *Aspergillus* antigens on the superficial cornea seems to allow consistently high fluorescence regardless of sample collection order. We observed similar signals in the donkey serum negative control samples and in negative control corneal samples. This leads us to believe that the background signal is equivalent due to having used the same blocking step.

In the future, antibody design may allow more detailed analysis of infectious etiology. One may choose antibodies for virulence factors or antibodies assessing antifungal susceptibility. Moreover, non-specific antibodies capable of differentiating broad categories of etiologies may also be useful. An antibody which recognizes all common fungal pathogens could be envisioned.

Our ex vivo model replicates corneal infection conditions with three-dimensional fungal extension in both *Aspergillus* and *Candida* inoculations. In *Aspergillus*, we observed the formation of a mycelium on the epithelial surface of the cornea. In contrast, *Candida* growth remained mainly superficial. We note differences in the appearance of fungal propagation at higher magnification. In the fluorescent images we see shifts in the focal distances of hypha structure in both *candida* and *aspergillus* (Figs. 6B and 7B). These differences in focal distance likely correspond to depth of penetration of fungal hyphae. We observed more deep penetration in the propagation of *Aspergillus* (Fig. 7B). For *Candida*, on the other hand, we observe labeled circular punctate superficial fungal components which likely represent *Candida* spores (Fig. 6B). This behavior was mainly analyzed in our study through standard microscopy future studies with confocal microscopy better confirm these findings. Most importantly, our model replicated mycelium formation in *Aspergillus* and hyphal extension in *Candida* growth. This property of our model is essential for understanding behavior of fungus during infection as hyphal growth also corresponds with differences in antigen expression [5]. As *Candida* presents with predominantly yeast morphology in vitro, a realistic model of growth is even more important for generalizability of results [5, 32].

Since most fungal keratitis infections in the community are connected to risk factors such as trauma, dirty wounds, and contact lens use, creation of a scratch prior to fungal inoculation may have more closely approximated real corneal infections [1, 2]. In addition, our ex vivo model implementation required flipping of the corneas, an orientation which does not exist in vivo. Alternatives used by others such as intrastromal injection in corneal tissue may not replicate the natural course of fungal inoculation as fungal keratitis generally is associated with dirty wounds of the corneal epithelium. One possible change to our model is the addition of a corneal epithelial scratch prior to inoculation. Regardless, our model recreated the stromal hyphal extension observed in fungal keratitis pathogenesis. Lastly, we would have preferred to have more repeats in each condition. This was largely limited by availability of human research tissue. Despite this limitation we believe our results to be robust due to consistency due sample collection repeat in each condition.

## Conclusions

As a result of our findings about the *Aspergillus fumigatus* and *Candida albicans* behavior in corneal infection in this work, we envision our PES membranes based KITs as an additional technique for fungal recognition in Infectious keratitis. The sample collection, the KIT design and fluorescence quantification methods we describe have potential applications as an alternative methodology for conducting immunoassays. In addition, this methodology may be expanded for use with other fungal and non-fungal pathogens. Our ex vivo model of fungal keratitis may be adapted for other fungal and bacterial pathogens; these could serve as platforms for evaluating future development of diagnostics for corneal infections. Results in our study emphasize the potential of immunoassay based diagnostic systems in Infectious keratitis. Further, our findings present a viable alternative ex vivo model for development of infection detection systems. Future work may elaborate further on adapting our fungal recognition system into a format applicable in point of care diagnostics.

## Electronic supplementary material

Below is the link to the electronic supplementary material.

Supplementary Material 1

## Data Availability

No datasets were generated or analysed during the current study.
