# Supplementary material for: Fungal infection monitoring on corneal epithelium ex vivo model and its collection over polyethersulfone membrane for detecting Candida albicans and Aspergillus fumigatus
Source: Med Microbiol Immunol. 2025 Feb 7;214(1):9. doi: 10.1007/s00430-025-00820-8 (PMC11805772; doi:10.1007/s00430-025-00820-8)
Supplement: Supplementary file 1 — Supplementary Material 1 [file 430_2025_820_MOESM1_ESM.pdf]

# *Supplementary FIGURES*

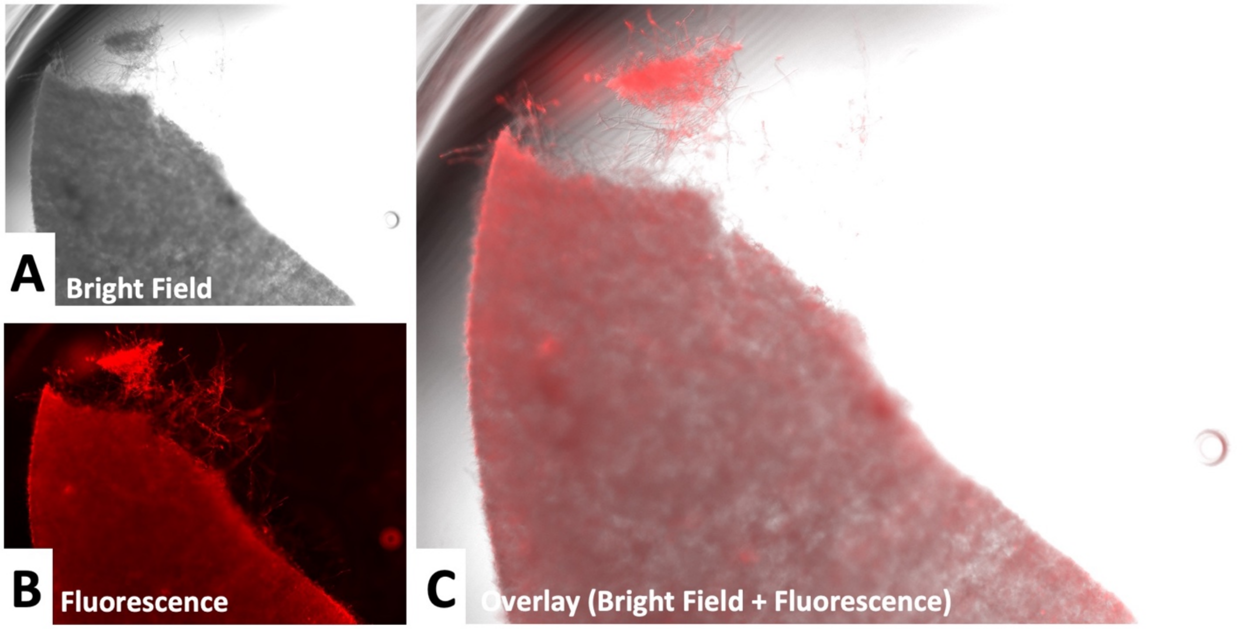


*Supplementary Figure 1: Aspergillus biofilm after removal from corneal surface: A. Bright field 4x, B.- Fluorescence 4x C. Overlay of fluorescence plus bright field. This figure demonstrates large hyphal propagation and biofilm formation properties of Aspergillus infections. This membrane is nearly completely composed of Aspergillus hyphal structures.*

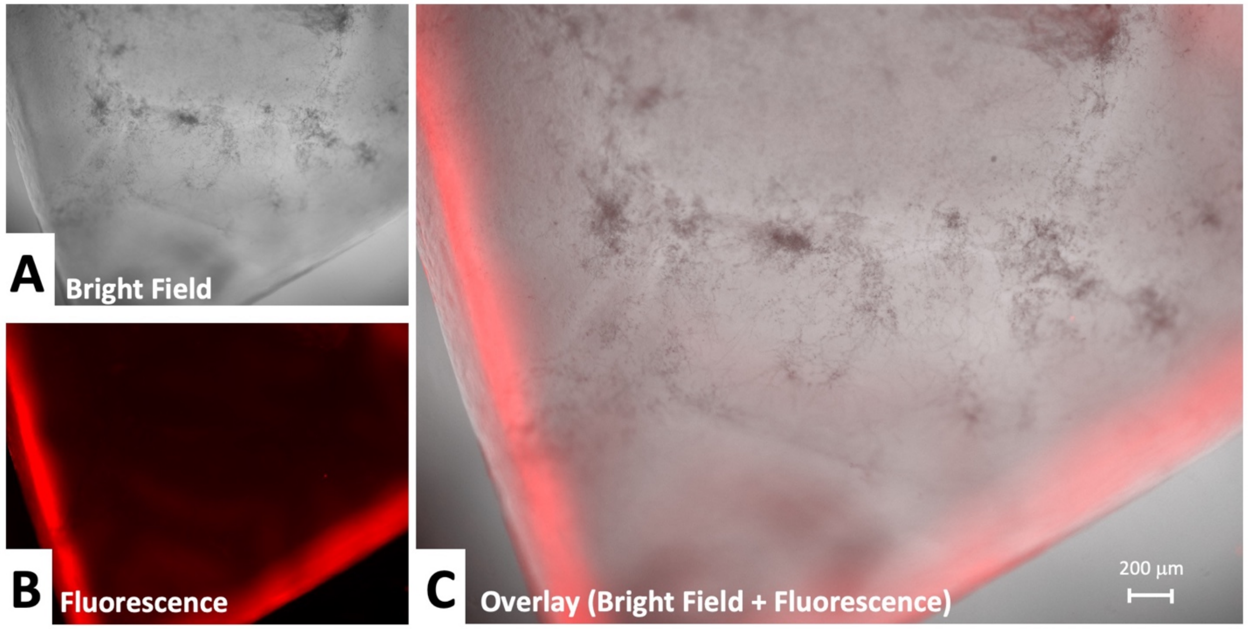


*Supplementary Figure 2: Candida inoculated cornea with incubation with Aspergillus specific primary antibody: A. Bright field 4x, B.- Fluorescence 4x C. Overlay of fluorescence plus bright field. This figure demonstrates the minimal to no cross reactivity of the primary Aspergillus antibody used with Candida fungal elements. Candida elements can be seen in the brightfield in the center of the image without fluorescent labeling. Some fluorescent background of the cornea can be observed at the edges.*

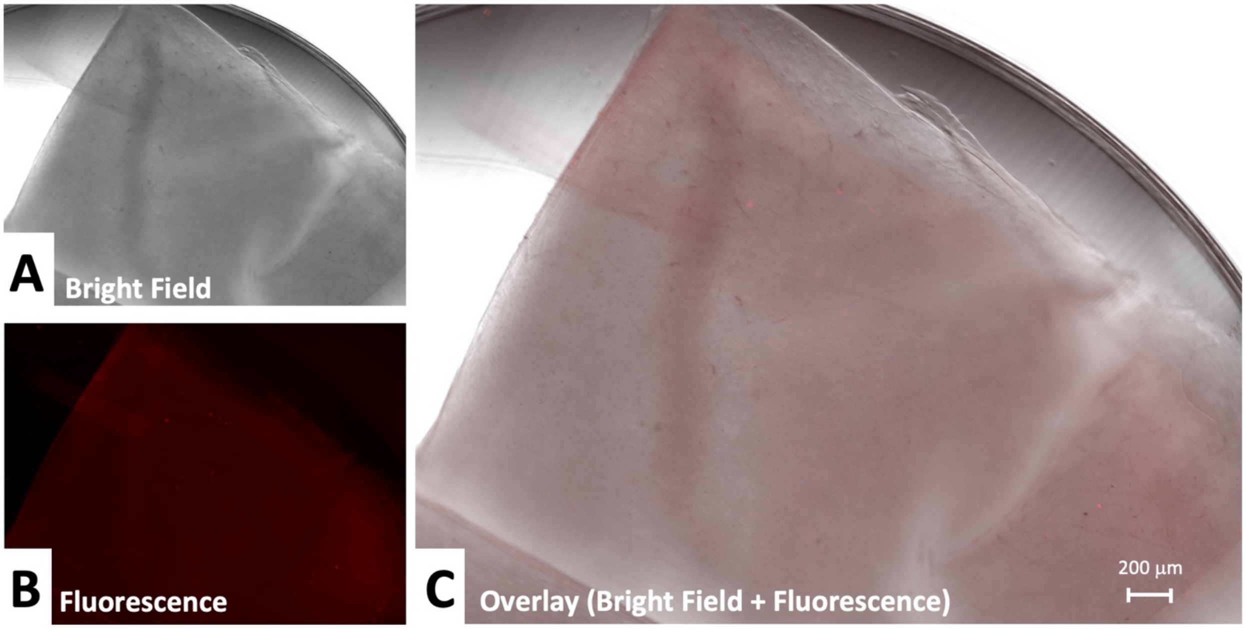


*Supplementary Figure 3: Aspergillus inoculated cornea with incubation with Candida specific primary antibody: A. Bright field 4x, B.- Fluorescence 4x C. Overlay of fluorescence plus bright field. This figure demonstrates the minimal to no cross reactivity of the primary Candida antibody used with Aspergillus fungal elements. Aspergillus elements can be seen in the brightfield in the image without fluorescent labeling. Minimal fluorescent background of the cornea can be observed diffusely.*

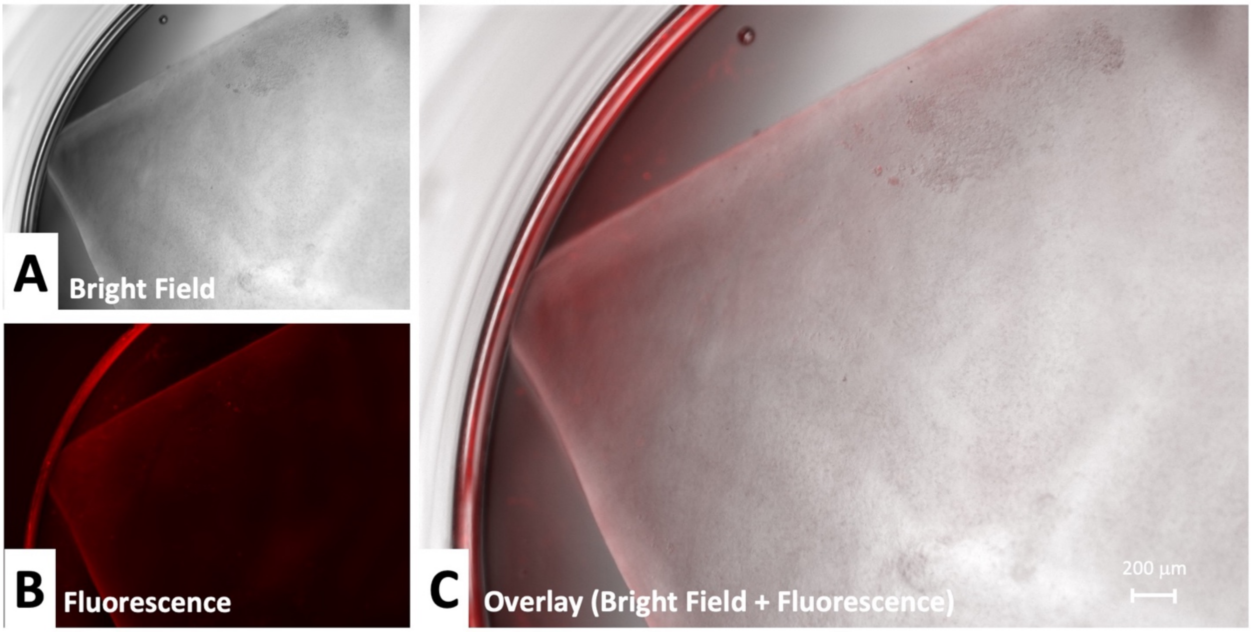


*Supplementary Figure 4: Negative control cornea with incubation in Candida specific primary antibody: A. Bright field 4x, B.- Fluorescence 4x C. Overlay of fluorescence plus bright field. This figure demonstrates the Candida antibody used demonstrates minimal affinity with control corneal elements at baseline. Corneal epithelial cells can be observed in brightfield on the corneal surface in the top right of the image.*

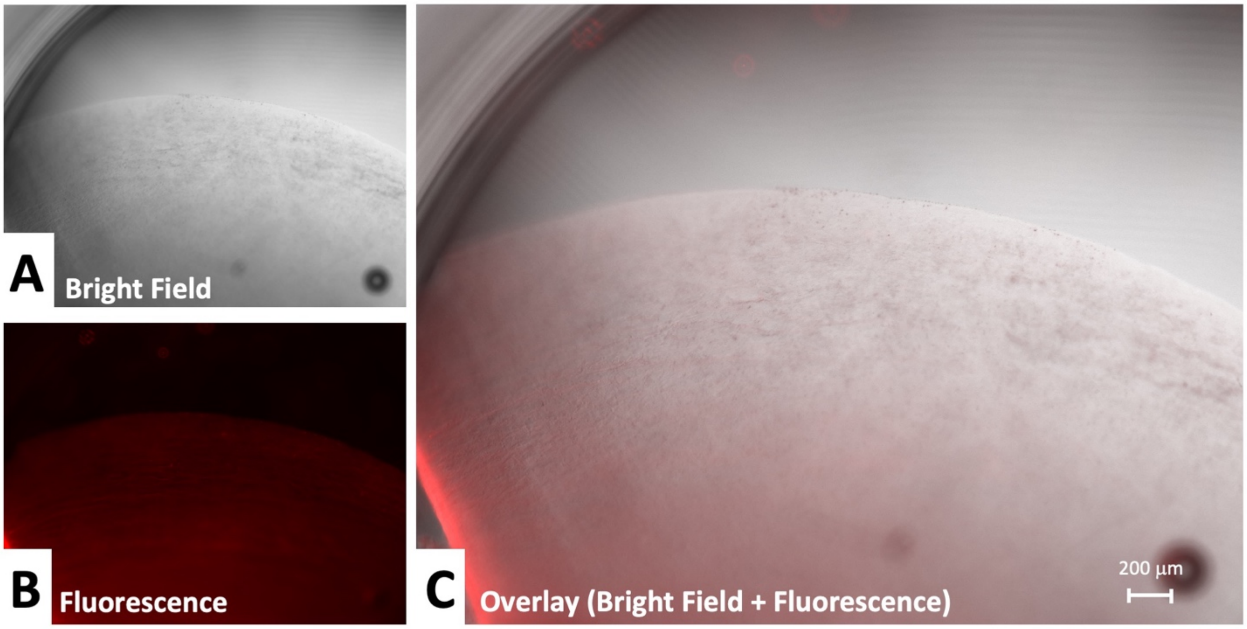


*Supplementary Figure 5: Control cornea with incubation with Aspergillus specific primary antibody: A. Bright field 4x, B.- Fluorescence 4x C. Overlay of fluorescence plus bright field. This figure demonstrates the Aspergillus antibody used demonstrates little reactivity with control corneal elements at baseline.*
